# Age- and Sex-Related Differences in Motor Performance During Sustained Maximal Voluntary Contraction of the First Dorsal Interosseous

**DOI:** 10.3389/fphys.2018.00637

**Published:** 2018-05-30

**Authors:** Valerie Sars, Roeland F. Prak, Tibor Hortobágyi, Inge Zijdewind

**Affiliations:** ^1^Department of Neuroscience, University of Groningen, University Medical Center Groningen, Groningen, Netherlands; ^2^Center for Human Movement Sciences, University of Groningen, University Medical Center Groningen, Groningen, Netherlands

**Keywords:** aging, sex differences, twitches, doublets, performance fatigability, contralateral activity, voluntary activation, FDI

## Abstract

Age and sex affect the neuromuscular system including performance fatigability. Data on performance fatigability and underlying mechanisms in hand muscles are scarce. Therefore, we determined the effects of age and sex on force decline, and the mechanisms contributing to force decline, during a sustained isometric maximal voluntary contraction (MVC) with the index finger abductor (first dorsal interosseous, FDI). Subjects (*n* = 51, age range: 19–77 years, 25 females) performed brief and a 2-min sustained MVC with the right FDI. Abduction force and root mean squared electromyographic activity (rms-EMG) were recorded in both hands. Double-pulse stimulation was applied to the ulnar nerve during (superimposed twitch) and after (doublet-force) the brief and sustained MVCs. Compared to females, males were stronger (134%, *p* < 0.001) and exhibited a greater decline in voluntary (difference: 8%, *p* = 0.010) and evoked (doublet) force (difference: 12%, *p* = 0.010) during and after the sustained MVC. Age did not affect MVC, force decline and superimposed twitch. The ratio between the doublet- and MVC-force was greater in females (0.33, *p* = 0.007) and in older (0.38, *p* = 0.06) individuals than in males (0.30) and younger (0.30) individuals; after the sustained MVC this ratio increased with age and the increase was larger for females compared to males (*p* = 0.04). The inadvertent contralateral, left force and rms-EMG activity increased over time (2.7–13.6% MVC and 5.4–17.7% MVC, respectively). Males had higher contralateral forces than females (*p* = 0.012) and contralateral force was higher at the start of the contralateral contraction in older compared with young subjects (difference: 29%, *p* = 0.008). In conclusion, our results suggest that the observed sex-differences in performance fatigability were mainly due to differences in peripheral muscle properties. Yet the reduced amount of contralateral activity and the larger difference in evoked versus voluntary force in female subjects indicate that sex-differences in voluntary activation should not be overlooked. These data obtained in neurological healthy adults provides a framework and help the interpretation and referencing of neurophysiological measures in patients suffering from neuromuscular diseases, who often present with symptoms of performance fatigability.

## Introduction

Performance fatigability is the decline in an objective measure of performance over a discrete period of time (Enoka and Duchateau, 2016). In the present study, we quantified performance fatigability by the decline in force during a sustained contraction. The details of the motor task, the muscles performing the task and subject characteristics are just a few of the variables that affect performance fatigability (Enoka and Stuart, 1992; Bigland-Ritchie et al., 1995; Allman and Rice, 2002; Hunter, 2016, 2017). Under most experimental conditions, performance fatigability is lower in women and older adults compared with younger men during isometric contractions at a similar percentage of maximal voluntary contraction (MVC) force (Hunter, 2014). Because sex- and age-effects are attenuated during high force isometric contractions (Hunter, 2014), most studies have examined performance fatigability using submaximal contractions of large muscles, including elbow flexors and knee extensors to detect age- and sex-differences (Hunter et al., 2004a; Mcphee et al., 2014; Solianik et al., 2017).

In contrast to large muscles, the small muscles of the hand are well suited for clinical studies focusing on (chronic) disease-related changes in neuromuscular function (Sheean et al., 1997; Thomas, 1997; de Ruiter et al., 2001; Thickbroom et al., 2006; Steens et al., 2012; Murray et al., 2014; Prak et al., 2015; Santarnecchi et al., 2015; Arnold et al., 2017). Previously we examined force decline during sustained maximal contractions with the index finger abductor muscle (the first dorsal interosseous, FDI). However, our sample sizes were small, the age ranges narrow, and the sex of the subjects inconsistently distributed for a systematic determination of the effects of age and sex on individual variation in voluntary and evoked forces and muscle activation. Because data concerning the effects of sex and age on these variables in hand muscles are scarce we decided to examine the effects of sustained MVCs on performance fatigability in the FDI of young and older individuals.

During an MVC, the contralateral homologous muscle typically becomes inadvertently active (Zijdewind and Kernell, 2001; Shinohara et al., 2003; Martin and Rattey, 2007; Post et al., 2008; Hortobagyi et al., 2011; Heetkamp et al., 2014). The magnitude of this activity, however, tends be higher in older and middle-aged compared with younger adults (Shinohara et al., 2003; Heetkamp et al., 2014). Although contralateral activity occurs in various tasks (Shinohara et al., 2003; Martin and Rattey, 2007; Post et al., 2008; Jiang et al., 2012; Watanabe et al., 2017) it is unclear if sex affects contralateral force and activation, parameters that are associated with effort and could be used as outcome variables to further characterize the nature and mechanism of performance fatigability.

The aim of this exploratory study was to determine the effects of age and sex on force decline during MVC, i.e., a measure of performance fatigability and on the mechanisms, using peripheral nerve stimulation, contributing to this force decline. The second aim was to determine the effects of age and sex on the magnitude of unintended force and activation produced in the contralateral, homologous FDI.

## Materials and Methods

Self-reported neurologically healthy subjects (age: 19–77, mean 48.84 ± 17.16 years, *n* = 51; 25 females) were included in the study. Some of these subjects were included as control subjects for earlier experiments (Steens et al., 2012; Prak et al., 2015). The University Medical Ethical Committee approved the protocol and the informed consent according to the guidelines of the Declaration of Helsinki (2013), which each subject signed prior to the start of the experiment.

All subjects completed the Fatigue Severity Scale (FSS), the Hospital Anxiety Depression Scale (HADS) and the Oldfield handedness questionnaires to assess perceived fatigue, mood and handedness, respectively (Oldfield, 1971; Zigmond and Snaith, 1983; Krupp et al., 1989).

### Experimental Setup

**FIGURE 1:**
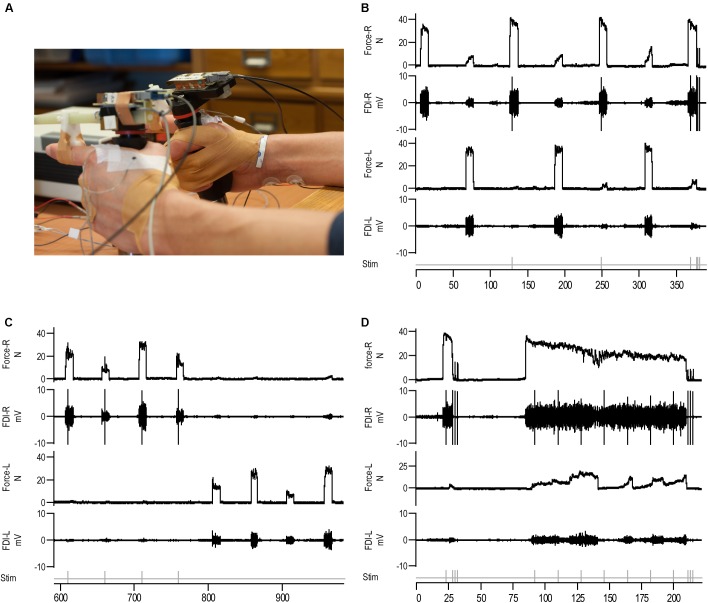
Illustration of the force transducer and an overview of the motor tasks as illustrated by data of an individual subject (female, 64 years old). **(A)** Photo of the hands holding the force transducers. EMG electrodes were taped to the muscle belly of the FDI and close to the second metacarpal joint. Stimulation electrodes were placed on the ulnar nerve of the right forearm, one electrode at and a second electrode approximately two centimeters proximal from the wrist. **(B)** An example of four right and three left maximal voluntary contractions (MVCs) at baseline. Subjects alternated between the right and left hand. Electrical stimulation (stim) was applied to the ulnar nerve during the MVCs with the right hand and at rest (during the first stimulus the subject was not at rest yet). **(C)** Submaximal contractions with the left and right FDI at four different levels (10, 30, 50, and 70% MVC). During each contraction with the right FDI, electrical stimulation was applied to the ulnar nerve. **(D)** Brief MVC with electrical stimulation of the ulnar nerve, followed by two to three stimuli at rest. After 60 s rest, subjects performed a sustained MVC for 124 s. During this sustained contraction seven stimuli were applied to the ulnar nerve (18 s interval). Following the sustained contraction three doublet-forces were evoked at rest.

Subjects were seated in a chair with both arms resting on a table adjustable for height with the elbow flexed at approximately 135°. Subjects were asked to place the forearms in a position midway between pronation and supination, so that abduction of the index finger occurred in a vertical direction, but their arm and the hand were not affixed to the table and remained free to move (see **Figure 1A**). A computer monitor was placed approximately 1 m in front of the subject providing visual feedback of the target force throughout the experiment.

Index finger abduction force was measured with custom-made force transducers (van Duinen et al., 2007, **Figure 1A**). The subjects held one transducer in each hand with the index fingers extended. The transducer was positioned such that a bar equipped with strain gages was parallel to the index finger; a C-shaped wedge placed over the proximal interphalangeal joint of the index finger connecting the force transducer to the index finger. To maintain the hand position in relation to the force transducer throughout the experiment, the thumb was taped to digit 3–5 and the index finger was taped to the C-shaped wedge on the transducer. The force signal was amplified and sampled for off-line analysis (CED 1401plus interface, Cambridge Electronic Design, United Kingdom; sampling frequency 500 Hz). During the experimental tasks, subjects could move each arm freely while holding the force transducer. However, the position of the hand in relation to the force transducer was constant.

### EMG Recordings

Electromyographic (EMG) activity was recorded bilaterally from the main index finger abductor, the FDI. After cleaning the skin with alcohol, two 2 mm surface electrodes (Ag-AgCl) with conducting gel were placed over the belly of the FDI and close to the second metacarpophalangeal joint (**Figure 1A**). A strap electrode was placed around the wrist of the right hand and served as a reference. The EMG signals were amplified (200x) and filtered (band pass filter: 10–1000 Hz) with a custom made amplifier, and sampled (at 2 kHz) using Spike2 (version 7.12b for Windows).

### Nerve Stimulation

Two electrodes were placed over the right ulnar nerve, one electrode at the wrist and a second electrode approximately two centimeters proximal to the wrist (**Figure 1A**) for electrical stimulation (DS7, Digitimer, United Kingdom) of the ulnar nerve. The ulnar nerve was stimulated with electric current (stimulus duration: 200 μs) starting from 5 mA with increasing steps of 5 mA to obtain a maximal EMG-response from the right FDI (Mmax). The constant-current stimulator could use a source voltage variable from 100 to 400 Volt to produce the necessary output current. Throughout the experiment the ulnar nerve was stimulated at ≥130% of the intensity needed to obtain Mmax (mean 39 mA, range: 25–67 mA).

To assess contractile properties and voluntary activation of the right FDI double-pulse stimulation (10 ms interstimulus interval) was given to the right ulnar nerve at rest and during voluntary contractions (superimposed twitch technique). The force evoked by the double-pulse stimulation at rest is referred to as doublet-force.

### Motor Tasks

Throughout the experiment subjects were encouraged to produce a maximal effort during the MVCs, no explicit instructions were given to the subject regarding the contralateral hand. During the first task, subjects were asked to produce seven MVCs, starting with the right FDI (4 MVCs) and alternating between hands (10 s duration, followed by 50 s rest, **Figure 1B**). During at least two right-hand MVCs, superimposed doublets (SITs) were evoked. After the last MVC two to three potentiated doublets at rest were evoked (some subjects maintained the contraction a little too long resulting in only two doublets being evoked at rest).

To evaluate differences in voluntary activation with a different method the second task consisted of three sets of submaximal efforts at 10, 30, 50, and 70% MVC in pseudo-random order, alternating sets between the left and right hand (**Figure 1C**). To determine the relationship between voluntary force and SITs (De Serres and Enoka, 1998), electrical stimulation was applied to the right ulnar nerve during at least two sets of submaximal contractions (minimal eight contractions).

The final task consisted of a brief MVC (6 s) followed by a 124 s sustained MVC (**Figure 1D**). Throughout the contraction, subjects were continuously encouraged to give their maximal effort. During the brief MVC, a SIT was evoked followed by three doublets at rest (pre-sustained doublets). In total, seven SITs were evoked during the sustained contraction (stimulus interval 18 s), followed by two or three doublets at rest immediately after the sustained contraction (post-sustained doublets, **Figure 1D**).

### Outcome Measures

During MVCs, peak force was measured and highest force of all MVCs was used as reference MVC. The EMG signals were root mean squared (rms) over 500 ms and the highest value was used as a reference for the EMG values. For the electrically evoked doublets the amplitude, contraction time (CT) and half relaxation time (HRT) were measured. The amplitude of the largest doublet at rest was used as a reference, for the CT and HRT we took average values as a reference.

The SITs were used to quantify voluntary activation using the following equation:

$$VA(\%)=100\times \left[1-\frac{SIT}{largest\;doublet\;atrest}\right]$$

During the submaximal contractions, the force at the time of the stimulation was determined and expressed as percentage of MVC. The SITs were expressed as percentage of the reference doublet.

During the sustained MVC, mean force, standard deviation (SD) of the force and rms-EMG obtained in the right (target side) and left hand were calculated over 2 s windows. To quantify force variability the SD was divided by the average force. The average of the first and last 6 s of the sustained contraction were used to index initial and end rms-EMG and force values. We also calculated a doublet/MVC ratio for (1) the doublet before the sustained MVC divided by the initial sustained MVC force, and (2) the doublet after the sustained MVC and the end sustained force values.

The twitches evoked during the sustained contraction were corrected for fatigue-related changes in the muscle with the following equation, before VA was calculated (Schillings et al., 2003).

$$\begin{array}{l} Corrected\;twitch(T)\\ =\frac{SIT\;(at\;t)}{presustained\;doublet-\left(\frac{t}{124S}\right)*(presustained-postsustained\;doublet)}*100 \end{array}$$

### Statistical Analysis

Primary outcomes were analyzed using SPSS (IBM SPSS Statistics, version 24). For outcome measures and residuals, normality assumptions were examined by probability and normality plots. If assumptions were violated, data were transformed.

Force and rms-EMG values obtained during the MVCs, and the initial and end force and rms-EMG values during the sustained contraction, were analyzed with univariate analysis of variance (ANOVAs) with sex as between subject factor and age as covariate. A repeated measures ANOVA (rm-ANOVA) was used to evaluate changes in doublets and doublet/MVC ratio with time (at baseline, prior to, and after the sustained MVC) as within subjects factor, sex as between subject factor and age as a covariate, including the interaction effect between sex and age.

SITs during submaximal contractions were analyzed using multilevel analysis (MLwiN for Windows, version 3.00, Centre for Multilevel Modelling, University of Bristol), with force (% MVC) as repeated measure at level 1 nested within subject at level 2 hierarchy. First, the model was fit using voluntary force (% MVC) as explanatory variable. Subsequently, intercept and slope were allowed to vary at both level 1 and level 2. Next, the fixed factors age, sex and their interaction terms were added. After each inclusion, the models were evaluated and only models with the smallest log-likelihood values survived.

To characterize changes over time within and between subjects we used pre-planned multilevel analysis for force, rms-EMG and SITs evoked during the sustained contraction with time as a first level variable nested within subject (level 2). These models were used to assess the effects of time, age, and sex on the outcome variable. First, the model was fitted with time and polynomials of time as fixed factors. Subsequently, intercepts were allowed to vary across subjects, followed by a model that allowed random slopes. Additionally, age, sex, and their interaction with time were included in the model. Only models with the lowest log-likelihood values survived.

In addition, Spearman correlations coefficients were calculated to determine the relation between perceived fatigue and mood with the percentage of force decline, age and sex, during the sustained MVC.

Statistical significance was set at α of 0.05. In the text mean and SD values are given unless stated otherwise. For multilevel model comparisons, difference in log likelihood values (LLH) are presented.

## Results

### Baseline Measurements

All subjects performed the task as instructed. In one male subject the force of the left hand was not obtained due to a technical problem with the force transducer and in another female subject random noise affected the EMG recording of the left FDI. The affected parameters of these two sets of data were removed from the analyses.

**Table 1** shows the subject characteristics. According to Oldfield Handedness Questionnaire (Oldfield, 1971)), one male subject was left handed (mean laterally index: 87 ± 27). All subjects presented an FSS score < 4.5, indicative of no perceived fatigue (Bakshi et al., 2000, **Table 1**) and HADS score < 8, suggesting no signs of depression (Spinhoven et al., 1997). These questionnaires revealed no age nor sex effect (sex: FSS: Spearman’s ρ = 0.124, *p* = 0.386; H_DS: Spearman’s ρ = -0.125, *p* = 0.381; HA_S: Spearman’s ρ = 0.076, *p* = 0.597; age: FSS: Spearman’s ρ = -0.165, *p* = 0.248; H_DS: Spearman’s ρ = 0.126, *p* = 0.379; HA_S: Spearman’s ρ = 0.062, *p* = 0.667).

**Table 1:** Primary outcomes of study population, separately for different age groups and sex (mean ± SD).

Variables						
Age	<30		30–40		40–50		50–60		60–70		70–80	
Sex	M (*n = 3*)	F (*n = 5*)	M (*n = 5*)	F (*n = 5*)	M (*n = 4*)	F (*n* = 5)	M (*n = 4*)	F (*n = 3*)	M (*n = 5*)	F (*n = 3*)	M (*n = 5*)	F (*n = 4*)
Left MVC (N)	53.4 (13.3)	44.7 (7.6)	57.9 (12.3)	49.4 (12.0)	51.0 (12.8)	36.9 (6.3)	46.0 (7.8)	32.1 (5.8)	54.2 (13.4)	39.42 (2.75)	45.3 (11.2)	31.6 (6.8)
Right MVC (N)	46.0 (8.5)	31.83 (7.2)	43.4 (9.8)	34.1 (8.8)	41.2 (16.3)	30.7 (6.0)	35.6 (3.9)	28.7 (4.3)	47.7 (10.9)	37.0 (9.2)	42.4 (17.4)	30.3 (3.2)
Doublet-force (N)	11.9 (4.7)	10.69 (2.12)	12.1 (2.1)	10.9 (3.2)	10.1 (6.4)	10.2 (2.6)	9.1 (1.7)	10.0 (3.4)	15.8 (2.2)	13.4 (4.9)	15.8 (3.8)	9.6 (2.1)
Doublet/MVC	0.25 (0.06)	0.34 (0.04)	0.29 (0.07)	0.32 (0.03)	0.23 (0.05)	0.34 (0.12)	0.26 (0.06)	0.34 (0.07)	0.34 (0.06)	0.35 (0.05)	0.40 (0.10)	0.32 (0.05)
Voluntary activation (VA)	96.6 (3.4)	96.2 (2.4)	92.7 (10.7)	95.6 (2.2)	96.1 (2.2)	91.5 (5.1)	97.1 (3.6)	92.4 (9.4)	95.3 (2.5)	93.4 (6.7)	89.6 (9.3)	93.6 (2.8)
Contraction time (CT)	90.8 (14.8)	102.6 (16.1)	92.3 (26.7)	99.5 (14.1)	90.3 (17.4)	100.5 (14.2)	106.0 (12.3)	97.8 (10.3)	90.4 (4.5)	94.2 (8.9)	90.8 (14.3)	95.3 (10.9)
Half relaxation time (HRT)	84.2 (10.9)	95.6 (13.0)	78.9 (9.6)	101.5 (7.3)	95.7 (14.7)	91.2 (6.4)	84.0 (11.3)	90.0 (11.3)	85.9 (12.9)	105.4 (30.9)	84.9 (10.4)	93.7 (13.2)
Superimposed twitch (SIT)	3.5 (3.4)	3.8 (2.4)	7.3 (10.7)	4.4 (2.2)	3.9 (2.2)	8.5 (5.2)	2.9 (3.6)	7.6 (9.4)	4.7 (2.5)	6.6 (6.7)	12.2 (9.6)	6.4 (6.7)
FSS	2.6 (1.4–4.0)		2.5 (1.8–3.4)		2.8 (1.1–3.9)		1.9 (1.3–3.2)		2.6 (1.1–3.6)		2.3 (1.7–3.7)	
H_DS	0.0 (0.0–3.0)		0.0 (0.0–2.0)		1.0 (0.0–2.0)		1.0 (0.0–2.0)		1.0 (0.0–4.0)		0.0 (0.0–4.0)	
HA_S	4.5 (0.0–7.0)		4.0 (1.0–6.0)		2.0 (0.0–6.0)		3.0 (0.0–7.0)		5.0 (3.0–7.0)		4.0 (0.0–7.0)	
Handedness	95 (80–100)		100 (58–100)		100 (67–100)		100 (68–100)		84.5 (-60–100)		90 (80–100)	

*Age in years; Sex divided in male (M) and female (F); MVC, (brief) maximal voluntary contraction; FDI, first dorsal interosseous; MVCs are in N; doublets are referred to as potentiated doublet-forces at rest; Voluntary activation (VA) in percentage; Doublet contraction time (CT) in ms; Doublet half relaxation time (HRT) in ms; Superimposed doublet-force (SIT) as % of doublet-force at rest; FSS, Fatigue Severity Scale; H_DS, subdivision depression of the Hospital Anxiety Depression Scale; HA_S, subdivision anxiety of the Hospital Anxiety Depression Scale (FSS, H_DS and HA_S scores in median and range); Handedness expressed as Oldfield (Oldfield, 1971) laterally index in percentage (median and range).*

### Brief Maximal Contractions

Both right-hand voluntary force (MVC: males: 43.0 ± 11.2 N vs. females: 32.0 ± 6.6 N, *p* < 0.001) and evoked force (doublet: males 12.7 ± 4.2 N; females 10.7 ± 2.9 N, *p* = 0.049) were higher in males than females. Larger MVCs were obtained in subjects with greater VA (mean: 94.1 ± 5.6%, *r* = 0.34, *p* = 0.016) but the VA did not differ between sexes (*p* = 0.83). The contraction time of the doublet did not differ [males: 93 ± 16 ms; females: 99 ± 13 ms; *F*_(1,47)_ = 1.873, *p* = 0.177], whereas half relaxation time (HRT) did differ between males (85 ± 12 ms) and females [96 ± 14 ms; *F*_(1,47)_ = 9.180, *p* = 0.004].

We found no main effect of age for MVC [*F*_(1,49)_ = 0.074, *p* = 0.787], voluntary activation [*F*_(1,49)_ = 2.012, *p* = 0.162], contraction time [*F*_(1,49)_ = 0.017, *p* = 0.90] or HRT [*F*_(1,49)_ = 0.013, *p* = 0.91].

**FIGURE 2:**
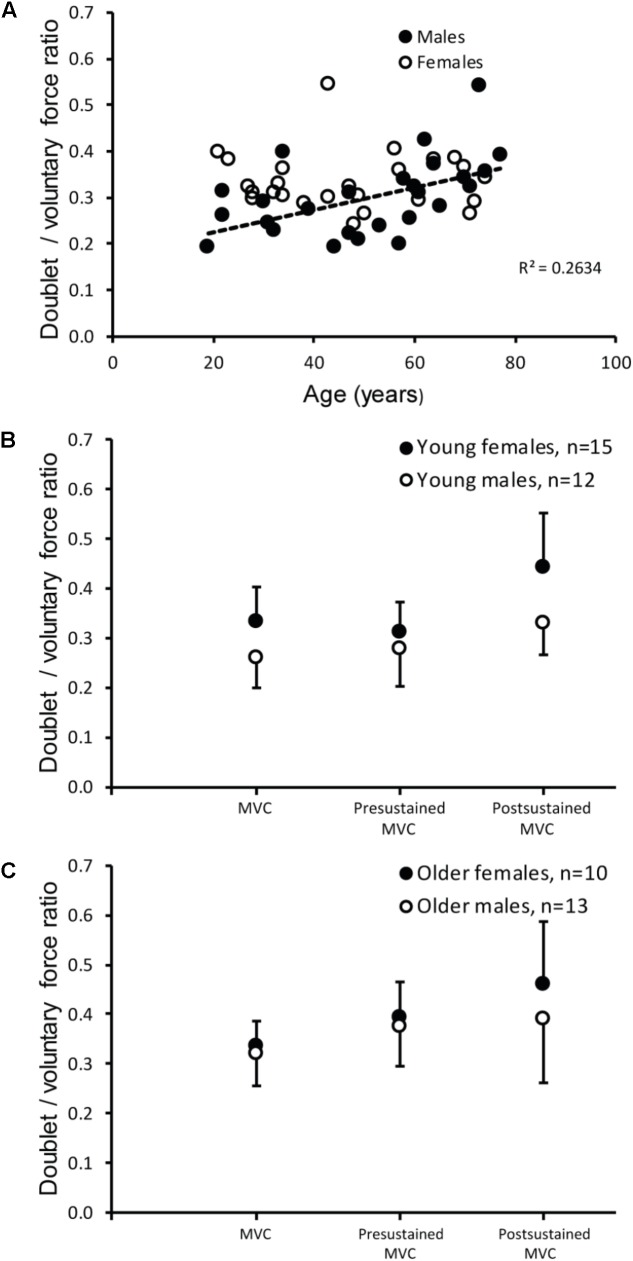
Mean and standard deviation of the doublet/voluntary force ratios. **(A)** Association between age and the ratio between the doublet at baseline and the MVC during brief MVC for male (closed symbols) and female subjects (open symbols). Data showed a significant effect of sex, and an interaction effect of sex ^∗^ age. **(B,C)** The doublet at baseline/MVC, doublet prior to the sustained MVC/mean force during first 6 s of sustained MVC (Presustained MVC), doublet after the sustained MVC/mean force during last 6 s of sustained MVC (Postsustained MVC). Data **(B)** is illustrated for young males (<50 years old, open symbols) and females (closed symbols) and **(C)** for older males (≥50 years old, open symbols) and females (closed symbols).

The ratio between the evoked doublet and the voluntary MVC was lower in males (0.30 ± 0.08) than in females [0.33 ± 0.06; *F*_(1,47)_ = 7.991, *p* = 0.007, **Figure 2**]. Additionally, this ratio showed a trend for an effect of age [*F*_(1,47)_ = 3.716, *p* = 0.06] and an interaction effect of sex ^∗^ age [*F*_(1,47)_ = 5.316, *p* = 0.026; **Figure 2A**]. The interaction effect was mainly due to an increase in the doublet/MVC ratio in older males (0.022 increase per year).

The MVCs of the left hand also differed between male (51.6 ± 11.7 N) and female subjects (39.8 ± 9.7 N; *p* < 0.001). The MVCs obtained in the left and right hand were correlated (*r* = 0.75, *p* < 0.001), however, only the MVC of the left hand differed with age (*r* = -0.30, *p* = 0.017). Sex and age together explained 36% of the variability in MVC (*p* < 0.005).

While producing MVCs, the contralateral hand also showed an index finger abduction force (in left hand: median: 5.3% of MVC, range: 0.66–30.43%; in right hand: median 4.1% of MVC, range: 0.37–37.49%; *Z* = -1.3, *p* = 0.19). The associated force in the left hand was positively associated with age (Spearman’s ρ = 0.47, *p* = 0.001) but did not differ by sex.

### Submaximal Contractions

**FIGURE 3:**
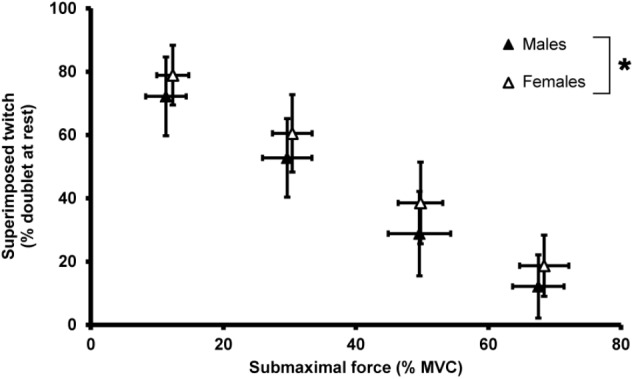
Mean and standard deviation of the doublet-force for males and females during different force levels of submaximal contractions. Submaximal contractions were performed at circa 10, 30, 50, and 70% of subjects’ MVC. Doublet-forces were normalized as percentage of maximal doublet-force at rest during baseline measurements. Closed symbols represent male subjects, open symbols represent female subjects. The asterisk identifies a significant difference between males and females.

To examine voluntary activation with a different method we evoked SITs during submaximal right hand index finger abductions. Multilevel analysis showed that the model improved (*p* < 0.001) after allowing random intercept and slopes for each individual. This model was further improved by including sex as explanatory variable (ΔLLH = 5.93, *p* = 0.007; sex: *Z* = 2.96; *p* = 0.003). The sex ^∗^ force interaction did not improve the model further, demonstrating that the difference in SIT did not depend on the force levels (**Figure 3**). Adding age as a fixed variable did not further improve the model (*p* > 0.05).

### Sustained Maximal Voluntary Contraction

**FIGURE 4:**
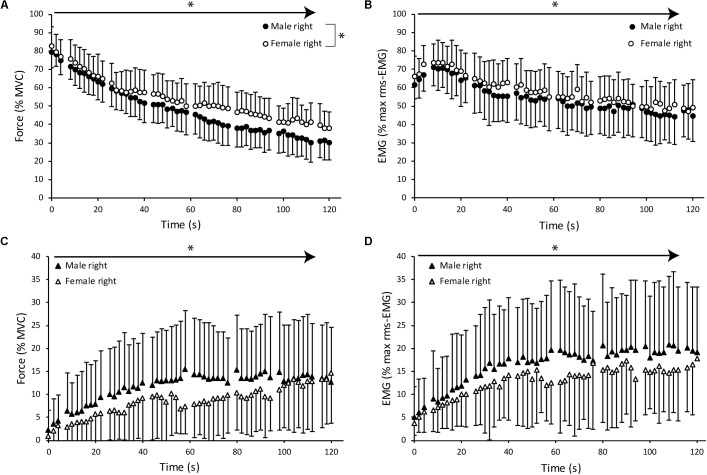
Mean and standard deviation of index finger abduction force and root mean squared EMG (rms-EMG) of the first dorsal interosseous (FDI) averaged over 2 s windows during the sustained MVC for males and females. Abduction force expressed as percentage of maximal force and rms-EMG activity expressed as percentage of maximal rms-EMG during brief MVCs at baseline. Values obtained at the times of peripheral nerve stimulation were excluded (every 18 s). **(A)** Index finger abduction force obtained in the right hand for male (closed symbols) and female subjects (open symbols). **(B)** Mean rms-EMG activity of the right FDI for male and female subjects during sustained MVC. **(C)** Averaged index finger abduction force obtained from the left non-target hand. **(D)** Mean rms-EMG activity of the left, contralateral non-target FDI for male and female subjects separately. The arrows with asterisks identify significant changes over time. Additionally, the significant interaction effect of time ^∗^ sex is identified with a bracket and asterisk.

**FIGURE 5:**
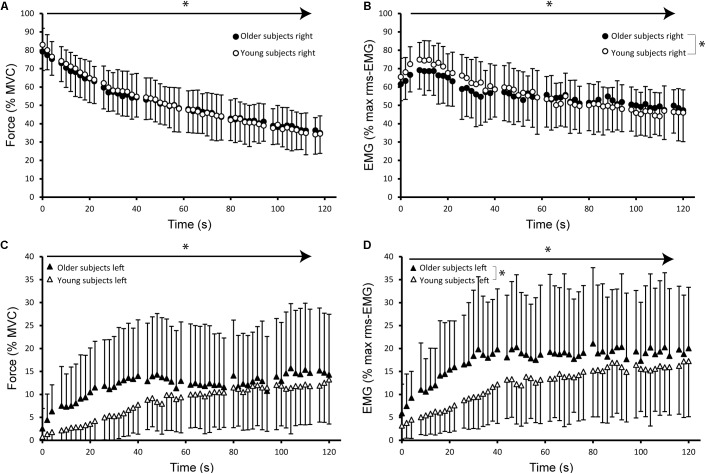
Mean and standard deviation of index finger abduction force and rms-EMG of the FDI averaged over 2 s windows during the sustained MVC for young (<50 years old, *n* = 27, 15 females; open symbols) and older (≥50 years old, *n* = 24, 10 female; closed symbols) subjects. Values obtained at the times of peripheral nerve stimulation were excluded (every 18 s). **(A)** Index finger abduction force obtained in the right hand during sustained MVC. **(B)** Averaged rms-EMG activity of the right FDI. Older subjects showed a smaller potentiation at the start of the sustained contraction, whereas young subjects show a gradual decline in rms-EMG activity of the right FDI. **(C)** Averaged index finger abduction force of the left hand. **(D)** Averaged rms-EMG activity of the left FDI. Older subjects exhibited higher averaged rms-EMG activity compared to young subjects. The arrows with asterisks identify significant changes over time. Additionally, the significant interaction effects of time ^∗^ age are identified with a bracket and asterisk.

For all parameters, the multilevel analysis revealed that the model with the best fit included time, and the 2nd and 3rd order polynomial of time, pointing to non-linear changes over time (**Figures 4**, **5**). The models improved further by allowing intercepts and slopes to vary randomly for each subject, illustrating the large variability between subjects. Throughout the results section, this model will be referred to as the basic model.

#### Right Index Finger Abduction Force

The brief MVC prior to the sustained MVC declined to 33.2 N [±9.6; 88% of initial MVC; *F*_(1,47)_ = 5.044, *p* = 0.029]. However, neither sex nor age affected this decline [sex: *F*_(1,47)_ = 2.653, *p* = 0.110; age: *F*_(1,47)_ = 0.414, *p* = 0.523].

Index finger abduction force declined further during the sustained MVC over time (*p* < 0.001). Mean force averaged over the first 6 s of the sustained contraction (79.1% MVC ± 7.97) was not different between males (78.1% MVC ± 7.04) and females (80.0% MVC ± 8.86; **Figure 4A**). However, at the end of the sustained contraction, the abduction force declined to 30.8% of MVC (±9.4%) in male and to 38.7% of MVC (±9.2%) in female participants [*F*_(1,48)_ = 7.1, *p* = 0.010].

Multilevel modeling confirmed this observation; adding sex and the interaction of sex ^∗^ time improved the basic model significantly [ΔLLH = 4.41, *p* = 0.015; sex ^∗^ time: *Z* = 2.27, *p* = 0.023]. We further evaluated whether differences in MVC could explain the effect of sex by including the right hand MVC and the interaction term of MVC ^∗^ time into the basic model. Adding these variables did improve the basic model but the comparison between this model and the model including sex demonstrated that the latter model was superior (ΔLLH = 6.72, *p* = 0.01). In contrast, no improvement was observed after adding age or the interaction of age ^∗^ time into the basic model (*p* = 0.496; *p* = 0.461, respectively; **Figure 5A**).

#### The rms-EMG of the Right FDI

The maximal rms-EMG during the brief MVC prior to the sustained contraction equaled 96.3% (23.5 SD) of the initial MVC. Mean 2-s rms-EMG values increased in the first 6 s from 66.3% (±8.0%) to 72.2% (±9.5%) of the initial rms-EMG and started to decline after 15 s to 47.3% (±13.0%) at the end of the sustained MVC (*p* < 0.001). The EMG activity at the end of the contraction did not differ between the sexes [*F*_(1,48)_ = 0.36, *p* = 0.549, **Figure 4B**] nor with age [*F*_(1,48)_ = 1.45, *p* = 0.234, **Figure 5B**]. The multilevel analysis also showed no main or interaction effect of sex on the EMG activity (ΔLLH = 1.31, *p* = 0.315). However, after inclusion of age and the interaction term of age ^∗^ time the model explained EMG variation significantly better (ΔLLH = 8.26, *p* = 0.0014; age ^∗^ time: *Z* = 3.64, *p* < 0.001). That is, older adults showed a smaller increase in EMG in the first minute of the sustained contraction than younger adults (**Figure 5B**) and a smaller decline in the second half of the sustained MVC.

#### Variability of Force

Force variability increased in both males and females from the start to the end of the sustained contraction (0.04–0.10, *p* < 0.001). Neither for the first 6 s nor for the last 6 s of the sustained contraction, did the force variability differ between males and females (*p* = 0.194; *p* = 0.743, respectively). The multilevel analysis also showed no effects of sex, age or the interaction terms of sex with time on EMG activity (although a trend was observed for age ^∗^ time; ΔLLH = 4.93, *p* = 0.079; *Z* = -2.059; *p* = 0.039).

### Associated Activity During the Sustained Maximal Voluntary Contraction

During right index finger abduction, force in the contralateral non-target hand progressively increased from 2.6% (±3.7%) to 13.6% of MVC (±10.8%, *p* < 0.001, **Figure 4C**).

Adding sex to the basic model explained more of the variance (ΔLLH = 4.89, *p* = 0.032; sex: *Z* = -2.50; *p* = 0.012). Age showed a tendency to improve the model (ΔLLH = 3.40, *p* = 0.065; age: *Z* = 1.952, *p* = 0.051, **Figure 5C**) without significant interaction terms. As can be observed in **Figure 4C**, the analysis implies that, overall, mean contralateral activity is larger in males (11.89 ± 9.12% MVC) than in females (8.6 ± 6.01% MVC).

The EMG activity of the left FDI showed no significant effect of sex (**Figure 4D**). Including age in the basic model contributed to a better model with a trend toward significance (ΔLLH = 3.073, *p* = 0.080; age: *Z* = 1.968, *p* = 0.049). Adding the interaction term of age ^∗^ time further improved the model (ΔLLH = 10.306, *p* = 0.006; age^∗^time: *Z* = -2.87, *p* = 0.004). **Figure 5** shows the larger EMG in the older than the younger subjects during the first minute.

### Doublet-Force Before, During and After the Sustained Contraction

#### Doublet-Force at Rest

Repeated measures ANOVA revealed that the doublet declined from pre- to post-sustained contraction [*F*_(1,64)_ = 195.58; *p* < 0.001]. Moreover, the interaction effect of doublet ^∗^ sex was significant [*F*_(1,64)_ = 9.66; *p* = 0.001], revealing that the decrease in doublet-force after the sustained MVC was higher in males (60.9 ± 13.4% of initial doublet) than females (49.0 ± 14.3% of initial doublet). The decline in doublet-force was associated with the decline in MVC (*r* = 0.48, *p* < 0.001).

The ratio of doublet and sustained MVC at the start (males: 0.33 ± 0.09, females: 0.34 ± 0.07) was smaller than the ratio at the end of the sustained MVC (males: 0.36 ± 0.11, females: 0.45 ± 0.11; **Figures 2B,C**). Statistical analysis of these ratios revealed main effects of time [*F*_(1,47)_ = 10.79, *p* = 0.002], sex [*F*_(1,47)_ = 7.17, *p* = 0.010], and age [*F*_(1,47)_ = 5.26, *p* = 0.026]. Additionally, an interaction effect of time and sex [*F*_(1,47)_ = 4.54, *p* = 0.038], and a trend toward a significant effect of time and age were found [*F*_(1,47)_ = 3.89, *p* = 0.055]. In other words, the doublet/voluntary force ratio was larger in females and in older subjects and the decline in doublet-force was less than the decline voluntary force in females after the sustained contraction. One subject was excluded from the analysis; the post-sustained ratio (1.41) differed more than 3 SD from the mean.

After the sustained contraction, the HRT of the doublet was increased but males (119.7 ± 26.6% of initial) still had shorter HRTs than females [124.0 ± 26.2% of initial; *F*_(1,49)_ = 7.401, *p* = 0.009]. Age, however, did not contribute to the increase in HRT or CT [HRT: *F*_(1,49)_ = 1.46, *p* = 0.233; although CT showed a trend toward significance CT: *F*_(1,49)_ = 3.83, *p* = 0.056].

#### Superimposed Twitches (SITs)

**FIGURE 6:**
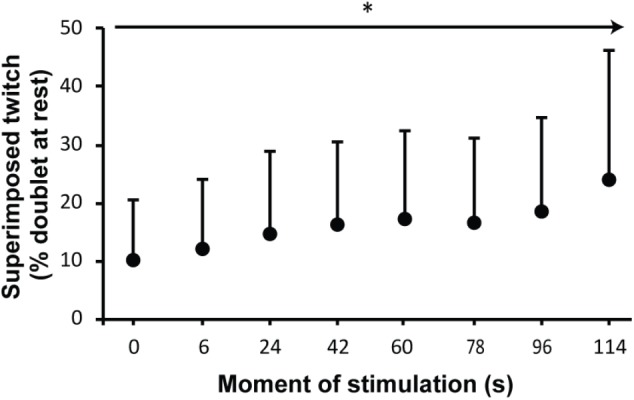
Mean and standard deviation of the superimposed twitches (SITs) as percentage of presustained twitch at rest. The *x*-axis shows times of stimulation; (0) SIT during brief MVC prior to the sustained MVC and times of SITs during sustained MVC (in seconds). The arrow with asterisk identifies a significant change over time.

The SITs during the sustained contraction were analyzed with multilevel analysis. In total, seven double stimuli were applied with an interval of 18 s. The model that explained the variance in SIT amplitude the best only included time, allowing random intercepts and slopes (**Figure 6**). Sex and age did not contribute to an improved model, which implies that the observed increase in SIT amplitude throughout the sustained MVC did not differ by sex or age.

#### Additional Analysis

Because our data suggested that changes in peripheral muscle properties were important contributors to the decline in voluntary force, we included doublet-force (% presustained doublet) in a model to explain the variance in voluntary force. This model explained force decline better than the model including sex (ΔLLH = 55.48, *p* < 0.001).

## Discussion

The primary finding of this study was that during a sustained maximal contraction of a small hand muscle, force declined more in males than females but without a difference in rms-EMG. This difference was independent of variation in MVC force but did depend on the decline in the electrically evoked doublet after the sustained contraction. Subjects showing a larger decline in the evoked force also showed a larger decline in voluntary force; both forces showed a larger decline in males. Nevertheless, this decline in doublet-force was, however, smaller than the decline in voluntary force, especially in females. Age, on the other hand, did not affect force decline but did affect rms-EMG, showing a smaller increase in the rms-EMG activity at the start of the contraction for older versus younger individuals. Throughout the contraction, the superimposed twitches increased independent of sex and age. Overall, the results suggest that the sex differences were mainly due to differences in peripheral muscle properties. This conclusion was further strengthened by additional analysis showing that changes in electrically evoked doublets explained variation in voluntary force even more accurately than sex differences. Yet the greater decline in voluntary than evoked force, especially in females, combined with less contralateral activity suggests that sex-differences in voluntary activation of the FDI should not be overlooked.

### Sex Differences During the Sustained Contraction

During the sustained MVC with the FDI, force declined less in females than males. This result is expected if one considers data obtained in other (larger) muscle groups during submaximal contractions (West et al., 1995; Russ and Kent-Braun, 2003; Hunter et al., 2004a; Senefeld et al., 2013; Ansdell et al., 2017; Solianik et al., 2017). However, this result is in contrast with data obtained in an intrinsic hand muscle, the adductor pollicis. During intermittent MVCs (Ditor and Hicks, 2000), electrically evoked 30-Hz contractions (Zijdewind and Kernell, 1994) and in older subjects (Cheng et al., 2003), no sex-related differences were observed in this hand muscle. Sex differences in fatigability, however, could be masked in the adductor pollicis because this muscle has a high percentage of type I fibers (80%, Johnson et al., 1973; Round et al., 1984); FDI: 57%, (Johnson et al., 1973). It is suggested that females are more fatigue resistant than males due to a greater proportional area of type I fibers (Kent-Braun et al., 2012; Hunter, 2014) and the observed longer HRTs for females (∼15% longer) in the present data support the suggestion of more type I fibers.

Beyond differences in muscle fiber type composition, higher absolute strength in males versus females is often suggested as a reason for the smaller force decline in females. Higher contractile forces tend to reduce blood flow, hence oxygen supply to the working muscles, resulting in accumulation of metabolic by-products which can interfere with contractile function (Barnes, 1980). However, the model including sex explained more variance in force decline than the model containing MVC. Additionally, the argument referring to differences in MVC forces between male and females is probably less relevant during maximal than during submaximal contractions because intramuscular pressure restricts blood flow as long as the contraction is maximal independent of its magnitude (Barnes, 1980; Petrofsky and Hendershot, 1984). We also found that the doublet declined more in males than in females (difference: 12%) but without sex-differences in voluntary muscle activation and that the decline in doublet could explain more of the variability in force decline than sex. Together these observations point to sex-related differences in performance fatigability being induced by changes in the muscle (possibly fiber type related) rather than differences in the activation of the muscle. Yet, the larger doublet during the submaximal contractions and the relatively large decline in voluntary force (61%) relative to the decline in doublet-force (49%) in females could point to possible sex differences in voluntary muscle activation (Russ and Kent-Braun, 2003; Martin and Rattey, 2007) which we were unable to capture with the superimposed twitch technique during MVCs (see Methodological Considerations). Earlier experiments also showed conflicting results concerning superimposed twitches evoked during MVCs and submaximal contractions in elbow flexor muscles (De Serres and Enoka, 1998).

### Age Differences During Sustained Maximal Contraction

In experiments with electrically evoked contractions in hand muscles, force decline in older compared to younger participants resulted in conflicting results. Force decline was found to be less (adductor pollicis: Narici et al., 1991) or more (adductor pollicis: Lennmarken et al., 1985). Whereas experiments using voluntary contractions produced either no difference (thenar muscles: Bemben et al., 1996) or reduced force decline (Chan et al., 2000; Ditor and Hicks, 2000) in older compared with younger participants, we found no effects of age on force decline, but observed small differences in the EMG response with age. At the beginning of the contraction, a larger increase in EMG was seen in young subjects. This increase in EMG could be due to an increase in motor unit firing rate and or to potentiation of the muscle fiber action potential (Zijdewind and Kernell, 1994; Zijdewind et al., 1999). An increase in motor unit firing rate could suggest that subjects did not contract maximally at the start of the sustained contraction despite continuous encouragement. However, the lack of age differences in voluntary force and voluntary activation suggests that the possible submaximal activation did not differ with age. The EMG potentiation is most likely a consequence of slowing of the muscle fiber action potential and hyperpolarization of the muscle fiber membrane (Hicks and McComas, 1989; Hicks et al., 1989) because of increased activation of the sodium-potassium pump. Whether this difference in potentiation reflects an age-related change in the activation of this pump during activity remains unclear. The smaller decline at the end of the sustained contraction accompanies the slightly smaller decline in force in older subjects.

The increase in the ratio between the doublet and MVC with age (∼2% per year) was unexpected. Similar to the larger ratio in females compared with males, it could reflect reduced activation during MVCs (Bilodeau et al., 2001; however, Chan et al., 2000; Solianik et al., 2017) but also age- and sex-related changes in the muscle stiffness could play a role (Eby et al., 2015). Increased muscle stiffness, as observed in older and female individuals (Eby et al., 2015), enhances the translation of contractile shortening into force production during short-lasting contractions.

An important difference between the present and previous experiments is that we searched for age-related changes with age as a continuous variable whereas most authors compared (two) groups of younger and older individuals. MVC force is relatively stable until over age 60 (Narici et al., 1991), although the decline in MVC force tend to be greater and earlier in males than females (Ditroilo et al., 2010; Abe et al., 2016). In our experimental group only a few individuals were 70 or more years of age (*n* = 9). In addition, the older adults in the current study were still physically active (most subjects cycled to our lab), experienced an overall wellbeing as illustrated by low scores on the FSS and HADS questionnaires, which could cause a bias toward the fit and healthy population of older adults.

### Contralateral Activity During the Sustained Maximal Voluntary Contraction

The unintentional activity in the contralateral FDI was higher in older compared with younger adults during both brief and at the start of the sustained contractions (Shinohara et al., 2003; Heetkamp et al., 2014). The time course of the contralateral force in older adults, i.e., more contralateral activity in older subject at the start of the contraction but a larger increase in young subjects agrees with previous data (Shinohara et al., 2003; Heetkamp et al., 2014). The amount of unintended contralateral activity increases with effort (Zijdewind and Kernell, 2001; Shinohara et al., 2003). The plateau phase at the end of the sustained contraction was not accompanied by a steeper increase in the superimposed twitches in the older subjects suggesting that the amount of effort was similar in young and older participants. It is suggested that the contralateral activity is the consequence of a reduced ability to suppress unwanted activity, possibly due to an age-related degeneration of the corpus callosum (Bashir et al., 2014). Another possible explanation is that older adults require additional and more widespread recruitment of brain areas, in order to compensate for neuromuscular changes with aging (Mattay et al., 2002; Naccarato et al., 2006).

No other study reported sex-differences in contralateral activity, but sex hormones can affect interhemispheric inhibition (Weis and Hausmann, 2010). Unfortunately, we did not collect data on the hormonal cycle of our female subjects. In addition to variation in sex hormones, effort could also affect contralateral activity. Although, few other experiments showed reduced activation in females (Martin and Rattey, 2007) most experiments did not observe differences between the males and females (Cheng et al., 2003; Hunter et al., 2006). Still, as discussed in earlier paragraphs, the submaximal data and the ratio between the doublet and voluntary force suggest that a difference in effort could play a role in the observed sex difference in force decline in the FDI.

### Methodological Considerations: Choice of Muscle and Contraction Intensity

Contrary to most (preclinical) studies, the present study examined sex- and age-differences during brief and sustained index finger abduction. The FDI contributes most to the index finger abduction and synergists minimally confound force output (An et al., 1983). A disadvantage is that stimulation of the ulnar nerve activates not only the FDI but also its antagonist (the second palmar interosseous, SPI; (An et al., 1983; Zijdewind and Kernell, 1994) which could result in an underestimation of the superimposed twitch and thus an overestimation of the voluntary activation. We reduced the contribution of the SPI by holding the force transducer in the hand (i.e., with fingers 3–5 flexed) with an extended index finger and the thumb adducted and taped to fingers 3–5 (**Figure 1A**). This position favors the length-tension relation of the FDI and reduces the contribution of the SPI. The relatively small contribution of the SPI at baseline is further illustrated by the large doublet/MVC ratio (0.32 ± 0.07). The relative contribution of the SPI increases during the sustained contraction but we feel that this would not affect males versus females or young versus older subjects differently. Furthermore, pilot data (*n* = 5) showed that the twitches evoked by ulnar stimulation and motor point stimulation resulted in similar estimates of index finger abduction force (see Discussion, Prak et al., 2015). The ratio between the maximal forces estimated on basis of the ulnar nerve stimulation and motor point stimulation equaled 0.95 (range 0.86–1.01) suggesting a relatively small contribution of the SPI. The intraclass correlation coefficient (absolute agreement) was 0.96 (*p* < 0.001).

We chose to use a sustained high intensity contraction, whereas most preclinical studies which focus on sex- and age differences used submaximal and/or intermittent contractions. For clinical populations (e.g., multiple sclerosis, spinal cord injury, mild traumatic brain injury), sustained maximal contractions are easier to standardize because they are less affected by problems with motor control (Severijns et al., 2017).

### Implications and Conclusion

The age- and sex-related effects on performance fatigability associated with a sustained maximal contraction we report here demonstrate the strength of including (male and female) subjects over a broad age range and provides a strong motivation for further longitudinal studies. Additionally, the observation that variables show (significant and non-significant) sex-specific changes with age stress the importance of population characteristics on basic muscle properties. Furthermore, this data is relevant to clinical populations. Such reference data are currently lacking but needed for a clearer distinction between changes in fatigability as the result of neuromuscular dysfunctions caused by disease and the (physiological) effects due to age and sex. The use of maximal, rather than submaximal, contractions in patients is related to the difficulty of standardizing the tasks. Heterogeneity between patients and clinical conditions in the levels of motor control and skills can confound performance fatigability produced in submaximal paradigms (Severijns et al., 2017). Yet studies in healthy adults often examine the effects of age and sex on performance fatigability caused by submaximal and intermittent contractions in large muscles (Enoka and Stuart, 1992; Allman and Rice, 2002; Hunter, 2016, 2017).

An implication of studying sex differences in individuals with a broad age range is that the interaction effects of age and sex can be ascertained. Also in this study (significant and non-significant) results showed that sex differences are more prominent in young compared to older individuals (Ditor and Hicks, 2000; Cheng et al., 2003; Hunter et al., 2004b).

In conclusion, we found that age- and sex-differences produced by sustained high intensity voluntary forces with a hand muscle are most likely caused by changes within the muscle. The age- and sex-related differences in performance fatigability in neurologically healthy adults help the interpretation and referencing of performance fatigability in persons suffering from neuromuscular disorders. Even in the absence of systematic effects of sex and age on performance fatigability these factors, if not controlled, could increase variability in outcome measures.

## Author Contributions

IZ: designed the experiment. VS, RP, and IZ: performed the experiments and analyzed the data. VS, RP, IZ, and TH: wrote the manuscript.

## Conflict of Interest Statement

The authors declare that the research was conducted in the absence of any commercial or financial relationships that could be construed as a potential conflict of interest.
